# Primary hyperparathyroidism incidence in northeastern Italy: A large population-based study

**DOI:** 10.1007/s40618-025-02723-0

**Published:** 2025-10-18

**Authors:** Simona Censi, Laura Salmaso, Giacomo Voltan, Filippo Ceccato, Ugo Fedeli, Maurizio Iacobone, Loris Bertazza, Cristina Clausi, Francesca Torresan, Caterina Mian, Mario Saia, Valentina Camozzi

**Affiliations:** 1 Department of Medicine (DIMED), University of Padua, Ospedale 105, 35121 Padua, Italy; 2 Endocrinology Unit, University Hospital of Padua, Padua, Italy; 3 Clinical Governance Unit, Azienda Zero Regione Veneto, Padua, Italy; 4 Endocrine Surgery Unit, Department of Surgical, Oncological and Gastroenterological Sciences (DiSCOG), University of Padua, Padua, Italy

**Keywords:** Hyperparathyroidism, Incidence, Population-based study, Epidemiology, Sex differences, Age distribution

## Abstract

**Purpose:**

Population-based studies of trends in the incidence of primary hyperparathyroidism (PHPT) in Italy were still lacking. Our aim was to estimate the incidence of PHPT in the Veneto region over the period 2012–2023.

**Methods:**

A retrospective, population-based study conducted in Veneto using the population registry and administrative health databases. Only subjects ≥ 18 years of age were included. We documented incident PHPT from 2013 to 2023 to exclude prevalent cases and calculated standardised incidence rates (IR) per 10,000 person-years by age and sex. We also made a sub analysis of only surgical cases.

**Results:**

We identified 5,276 incident cases (IR: 1.17). Overall, the incidence of PHPT was stable from 2013 to 2023, although there was a declining trend in males, at the limits of statistical significance with the standardised IR decreasing from 0.77 (95% CI: 0.64;0.91) in 2013 to 0.67 in 2023 (95% CI: 0.55;0.78) (p = 0.056). The overall female-to-male incidence ratio was 2.55, with females predominating, and reached a peak in the 50–54 age group (ratio: 3.94) and a nadir in 25–29 age group (ratio: 0.54). Median age was 65 years (IQR: 56–76 years) and was lower among females than males (64 *vs* 68 years) (p < 0.0001). Among the surgical cases, the percentage distribution of incident cases by age was almost the same for females and males, median age was similar (61 years for females, 62 years for males), while the rates remained higher for females than for males.

**Conclusion:**

Our study shows that over the past decade there has been a general stability in the incidence of PHPT in our Region. A higher incidence was observed in postmenopausal females and a different pattern of incidence emerged from our analysis of surgical cases.

**Supplementary Information:**

The online version contains supplementary material available at 10.1007/s40618-025-02723-0.

## Introduction

Among endocrine disorders, primary hyperparathyroidism (PHPT) ranks third in prevalence [1]. Over recent decades, the clinical profile of PHPT in developed countries has undergone a marked transformation from a condition predominantly impacting bones and kidneys to a largely asymptomatic disorder, frequently identified incidentally. The majority of cases are attributed to parathyroid adenomas (80–85%), with parathyroid hyperplasia responsible for 10–15%, and the rare parathyroid carcinoma accounting for the remaining 1–4% [2]. The broad accessibility of biochemical calcium testing in developed countries has played a key role in the transition from symptomatic to asymptomatic presentations of the disease [3]. In contrast, in developing regions, such as Asia and Africa, PHPT often presents with symptomatic skeletal or renal complications [4]. Differences in healthcare utilisation across countries have significantly influenced the prevalence and incidence of PHPT, both within individual regions and globally [5]. As a result, the incidence of PHPT remains poorly defined. The most reliable data from the UK [6], Denmark [7] and the USA [8, 9] indicate an annual incidence ranging from 4.0 to 95.7 cases per 100,000 inhabitants. This considerable variability stems from differences in screening techniques, case definitions, populations studied and annual fluctuations in incidence within the same population, an aspect that remains unexplained. However, the aforementioned incidence data were gathered more than a decade ago and as such are outdated. The clinical presentation of PHPT has been studied in a multicentric Italian study and has gathered many interesting data [10]. Other several recent studies, based on Italian case series, have sought to investigate the pathophysiological mechanisms, complications, and phenotype of PHPT in Italy [11–13]. However, to the best of our knowledge, no population-based data are available for Italy for any period.

Therefore, the aim of our study was to analyse the incidence trends of PHPT in the Veneto region, a high-income area with a comprehensive, publicly-funded health service [14], in the decade 2012–2023.

## Material and methods

### Study design

We conducted a retrospective population-based study in the Veneto region (northeastern Italy), which is divided into 7 provinces and 563 municipalities with a total population of about 4.9 million. The same methodology as in our two previous population-based studies was adopted [15, 16]. Healthcare services in Veneto are provided by nine local social and healthcare units, two university hospitals, two hospitals for scientific research and various accredited private providers, based on a hub-and-spoke model. Hospital care in Italy is free of charge for all residents and is covered by general taxation. The Veneto region is one of the most densely populated regions in the country (the fourth most populous), accounting for 8% of the entire Italian population and contributing 9.2% of Italy’s gross domestic product.

Inhabitants of the Veneto region between January 2012 and December 2023 were identified through a population registry, which has virtually full coverage. Residents were then linked with hospital discharge records and the healthcare co-payment exemption database. The latter includes information on all individuals with a diagnosis by a medical specialist of certain conditions for which the national health service provides specific free-of-charge outpatient services.

Hospital discharge records contain the following information for all inpatient episodes: (i) socio-demographic data (sex, age, place of residence, educational level); (ii) clinical data (primary and secondary diagnoses, any surgical or medical procedure performed on the patient, and the mode of discharge); and (iii) hospital data (hospital ward, admission and discharge dates).

All diagnoses are coded according to the International Classification of Diseases, Ninth Revision, Clinical Modification (ICD-9-CM) coding system, currently used in Italy.

All inhabitants of the Veneto region between 1 January 2012 and 31 December 2023 were included in the analyses. This time window was chosen as it was the period for which data were available to identify hyperparathyroidism cases in the registry. Residents with healthcare co-payment exemption for hyperparathyroidism (code 026 and ICD9CM diagnosis 252.0) or at least one hospitalisation between 2000 and 2023 with a primary diagnosis of hyperparathyroidism (ICD9CM 252.00—Hyperparathyroidism, unspecified; 252.01—Primary hyperparathyroidism) were considered affected by hyperparathyroidism. Because the electronic health record database for healthcare co-payment exemption was only established in 2012, we set 1 January 2013 as the starting point for our analyses, using the first year as a washout period to avoid misclassifying pre-existing (prevalent) cases of hyperparathyroidism as newly diagnosed (incident) cases. Only patients aged 18 years and above were included in the study. The incidence date was defined as the earliest of the dates of healthcare co-payment exemption or hospital discharge record.

In the incident cohort, patients undergoing surgery were identified by reviewing hospital admissions during the study period for the presence of the procedure codes 06.81—Total parathyroidectomy or 06.89—Other parathyroidectomy.

The cohort selection process is illustrated in Fig. 1 of the supplementary materials.

**Fig. 1:**
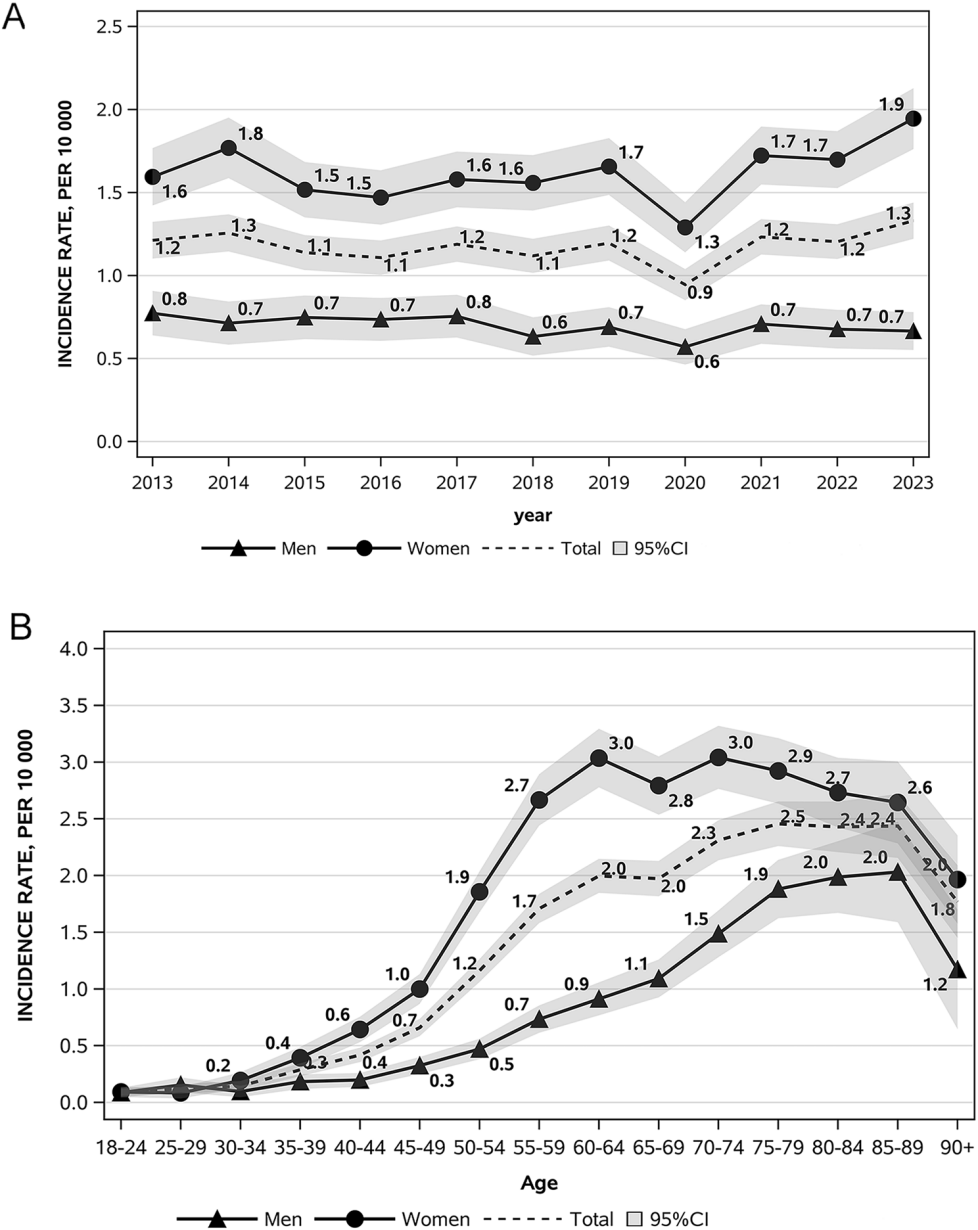
Standardised incidence rates by year and sex (**A**) and by age and sex (**B**)

### Measurements

The study was conducted using anonymised records comprising data routinely collected by healthcare services. All regional health records undergo a standardised anonymisation process that assigns a unique anonymous code to each individual, which allows for linkage between electronic health records without any possibility of back-retrieving the subject’s identity.

All data in the Local Health Authority registries are recorded with the patient’s consent and can be used as aggregate data for scientific studies without further authorisation (*Garante per la protezione dei dati personali*, Resolution n.85 of 1 st March 2012). This study complies with the Declaration of Helsinki and Italian Decree n.196/2003 on personal data protection.

### Patient and public involvement

No funds or time were allocated for patient and public involvement, so no patients were involved in the study.

### Statistical analysis

Univariate and bivariate analyses were performed to summarise the patients’ demographic data. Continuous variables are reported as descriptive statistics (mean, standard deviation (SD), median and interquartile range (IQR)), the categorical variables as frequencies and percentages. Differences between groups were ascertained by Student’s t-test or Mann–Whitney test for continuous variables, and Pearson’s chi-square test or Fisher’s exact test for categorical variables, as appropriate. A p-value < 0.05 was considered statistically significant. The yearly incidence rates (IR) were estimated by dividing the number of new patients diagnosed with hyperparathyroidism each year by the number of residents in the Veneto region in that year. Incidence rates per 10,000 people along with 95% confidence intervals (CI) were calculated for each year and stratified by age, sex and province of residence. Direct age-standardisation was performed using the resident population of the Veneto region in 2018 as the reference. The average annual percentage change (AAPC) in age-standardised rates and the relative 95% CI were obtained from linear regression models with the logarithm of age-standardised rates weighted by the inverse of their variance as the dependent variable and the corresponding year as the regressor.

As women may be diagnosed earlier due to routine screening, we calculated age- and sex-specific incidence rates not only for the whole cohort, but also just for patients who had undergone parathyroidectomy in order to capture those cases likely to have been diagnosed at more advanced stages.

All statistical analyses were conducted using the Statistical Analysis Software (SAS) version 9.4 (SAS Institute Inc., Cary, NC, USA).

## Results

During the study period, we identified 5,276 incident hyperparathyroidism cases, corresponding to a standardised incidence rate (IR) of 1.17 per 10,000 person-years (95% CI: 1.14;1.21). The median age at diagnosis was 65 years (IQR 56–76 years) and was lower among females than males (64 years, IQR 56–75 years *vs* 68 years, IQR 56–77 years; p < 0.0001). Overall, the incidence of hyperparathyroidism remained stable over the study period with a standardised IR of 1.21 (95% CI: 1.10,1.32) in 2013 and 1.33 in 2023 (95% CI: 1.22;1.44). However, males and females exhibited different trends. While in females the standardised IR remained largely stable, from 1.59 (95% CI: 1.42;1.77) in 2013 to 1.95 (95% CI: 1.76;2.13). in 2023 (p = 0.28), in males we observed a decreasing trend over the same period from 0.77 (95% CI: 0.64;0.91) in 2013 to 0.67 (95% CI: 0.55;0.78) in 2023 (AAPC: −1.51, 95% CI: −3.04;0.05, p = 0.056) (Fig. 1A and Table 1).

**Table 1:** Trends in standardised IR of hyperparathyroidism by sex in the Veneto Region between 2013 and 2023

Year			Male		Female	
	Incident cases (n)	Standardized IR (95%CI) per 10,000	Incident cases (n)	Standardized IR (95%CI) per 10,000	Incident cases (n)	Standardized IR (95%CI) per 10,000
2013	465	1.21 (1.10;1.32)	134	0.77 (0.64;0.91)	331	1.59 (1.42;1.77)
2014	489	1.26 (1.14;1.37)	122	0.71 (0.58;0.84)	367	1.77 (1.59;1.95)
2015	449	1.14 (1.03;1.24)	129	0.75 (0.62;0.88)	320	1.52 (1.35;1.68)
2016	442	1.11 (1.00;1.21)	130	0.74 (0.61;0.86)	312	1.47 (1.31;1.63)
2017	480	1.19 (1.08;1.30)	137	0.75 (0.63;0.88)	343	1.58 (1.41;1.75)
2018	457	1.12 (1.02;1.22)	119	0.63 (0.52;0.75)	338	1.56 (1.39;1.73)
2019	495	1.20 (1.09;1.30)	131	0.69 (0.57;0.81)	364	1.66 (1.49;1.83)
2020	395	0.94 (0.85;1.04)	111	0.57 (0.46;0.68)	284	1.29 (1.14;1.44)
2021	518	1.23 (1.13;1.34)	139	0.71 (0.59;0.83)	379	1.72 (1.55;1.90)
2022	514	1.20 (1.10;1.31)	132	0.68 (0.56;0.79)	382	1.70 (1.53;1.87)
2023	572	1.33 (1.22;1.44)	133	0.67 (0.55;0.78)	439	1.95 (1.76;2.13)
**Total**	**5276**	**1.17 (1.14;1.21)**	**1417**	**0.69 (0.66;0.73)**	**3859**	**1.62 (1.57;1.67)**

**Fig. 2:**
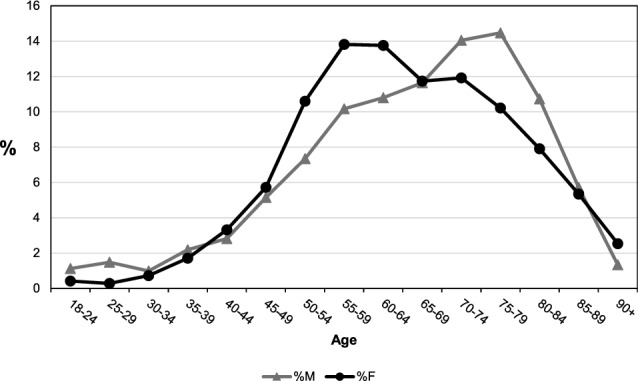
Percentage distribution of cases by age and sex

**Fig. 3:**
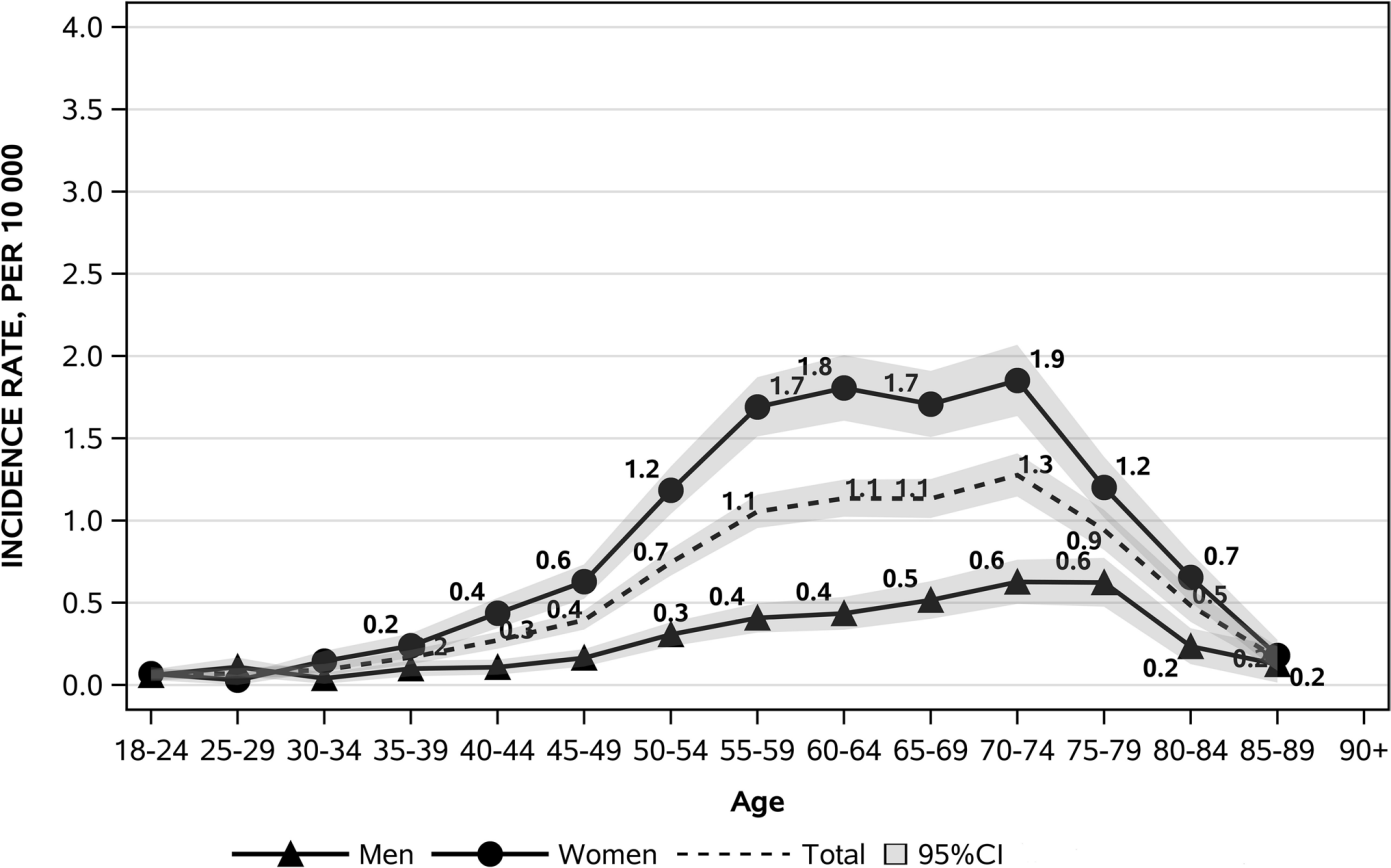
Age- and sex-specific incidence rates in patients having undergone a parathyroidectomy

**Fig. 4:**
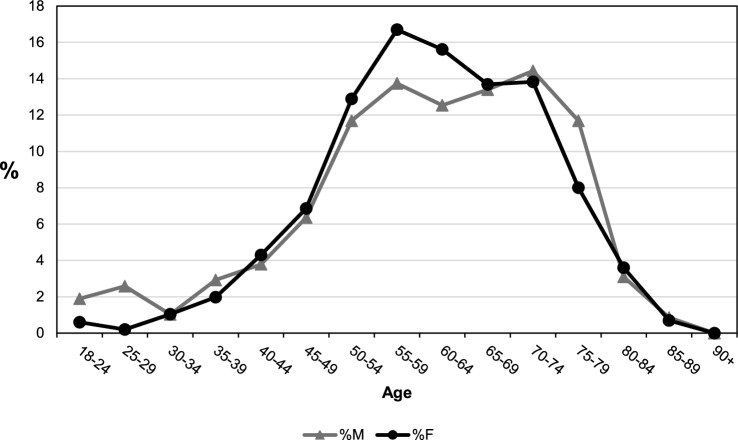
Percentage distribution of cases by age and gender in patients having undergone a parathyroidectomy

An out-of-trend decrease in hyperparathyroidism incidence was documented in 2020, corresponding to the SARS-CoV-2 pandemic (standardised IR 0.94, 95% CI: 0.85;1.04), with a realignment to the stable trend in the subsequent years (Fig. 1A and Table 1). Age-specific rates are significantly and consistently higher in females than in males from the age of 35 years upwards (Table 2). The female-to-male incidence ratio was 2.55 with females predominating, and reached a peak in the 50–54 age group (female-to-male ratio 3.94 p < 0.001) and a nadir in the 25–29 age group (female-to-male ratio of 0.54 p = 0.099), the latter the only age group where males predominated (Table 2 and Fig. 1B). Interestingly, the percentage distribution of cases based on sex and age showed a frequency peak 20 years earlier in females than in males (55–59 years *vs* 75–79 years) (Fig. 2). We carried out further analyses of the data taking into consideration only those patients who had undergone a parathyroidectomy in order to ascertain more precisely the incidence of symptomatic or more severe cases. Age-specific rates remained higher in females than in males in all age groups from 30 years, with crude rates of 0.88 per 10,000 person-years (95% CI: 0.83;0.90) and 0.27 per 10,000 person-years (95% CI: 0.25;0.29), respectively, and a female-to-male ratio of 3.26 (95% CI: 2.97;3.58) (Fig. 3). The female-to-male ratio peaked in the 60–64 age group (4.1495, p < 0.001), and the nadir remained in the 25–29 age group, where the IR was higher in males (female-to-male ratio of 0.28 p = 0.022) (Table 3). However, the median age at diagnosis was substantially the same at 61 years for females (IQR: 54–70 years) and 62 years for males (IQR: 52–71 years) (p = 0.495) and there was also a substantial change in the percentage distribution of cases based on sex and age (Fig. 4). The percentage distributions of incident cases by age were almost the same for the two sexes, and the curves of the age-specific rates were more symmetrical. However, the incident rates remained higher for females than for males, particularly over 55 years of age.

**Table 2:** Hyperparathyroidism IR by age and sex and the female-to-male IR ratio in the Veneto Region

Age group (years)	Incident cases (n)		IR (95%CI) per 10,000		Ratio F/M (95%CI)	p-value
	Male	Female	Male	Female		
18–24	16	16	0.09 (0.04;0.13)	0.09 (0.05;0.14)	1.07 (0.54;2.14)	0.845
25–29	21	11	0.15 (0.09;0.22)	0.08 (0.03;0.13)	0.54 (0.26;1.12)	0.099
30–34	14	28	0.10 (0.05;0.15)	0.19 (0.12;0.27)	2.02 (1.06;3.83)	0.032
35–39	31	66	0.18 (0.12;0.25)	0.39 (0.30;0.49)	2.15 (1.40;3.29)	0.000
40–44	40	128	0.20 (0.14;0.26)	0.64 (0.53;0.75)	3.26 (2.28;4.64)	0.000
45–49	73	221	0.32 (0.25;0.40)	1.00 (0.87;1.13)	3.08 (2.36;4.01)	0.000
50–54	104	409	0.47 (0.38;0.56)	1.86 (1.68;2.04)	3.94 (3.18;4.89)	0.000
55–59	144	533	0.73 (0.61;0.85)	2.67 (2.44;2.89)	3.63 (3.02;4.37)	0.000
60–64	153	531	0.91 (0.77;1.06)	3.04 (2.78;3.29)	3.33 (2.78;3.98)	0.000
65–69	165	453	1.09 (0.93;1.26)	2.79 (2.54;3.05)	2.56 (2.14;3.06)	0.000
70–74	199	460	1.49 (1.28;1.69)	3.04 (2.76;3.32)	2.05 (1.73;2.42)	0.000
75–79	205	394	1.88 (1.62;2.14)	2.92 (2.63;3.21)	1.55 (1.31;1.84)	0.000
80–84	152	305	1.99 (1.67;2.30)	2.73 (2.42;3.04)	1.37 (1.13;1.67)	0.001
85–89	81	206	2.03 (1.59;2.47)	2.64 (2.28;3.01)	1.30 (1.01;1.68)	0.044
90 +	19	98	1.17 (0.64;1.70)	1.96 (1.58;2.35)	1.68 (1.03;2.74)	0.039
Total^a^	1417	3859	0.65 (0.62;0.68)	1.66 (1.61;1.71)	2.55 (2.40;2.71)	0.000

^a^Sex-specific and overall crude incidence rates

**Table 3:** IR by age and sex and female-to-male IR ratio in patients having undergone Parathyroidectomy

Age group (years)	Incident cases (n)		IR (95%CI) per 10,000		Ratio F/M (95%CI)	p-value
	Male	Female	Male	Female		
18–24	11	12	0.06 (0.02;0.09)	0.07 (0.03;0.11)	1.17 (0.52;2.65)	0.708
25–29	15	4	0.11 (0.05;0.17)	0.03 (0.00;0.06)	0.28 (0.09;0.83)	0.022
30–34	6	21	0.04 (0.01;0.07)	0.15 (0.08;0.21)	3.53 (1.42;8.74)	0.006
35–39	17	40	0.10 (0.05;0.15)	0.24 (0.16;0.31)	2.37 (1.35;4.18)	0.003
40–44	22	87	0.11 (0.06;0.15)	0.44 (0.35;0.53)	4.02 (2.52;6.42)	0.000
45–49	37	139	0.16 (0.11;0.22)	0.63 (0.52;0.73)	3.82 (2.66;5.49)	0.000
50–54	68	261	0.31 (0.23;0.38)	1.18 (1.04;1.33)	3.85 (2.95;5.03)	0.000
55–59	80	338	0.41 (0.32;0.50)	1.69 (1.51;1.87)	4.15 (3.25;5.29)	0.000
60–64	73	316	0.44 (0.34;0.54)	1.81 (1.61;2.01)	4.15 (3.22;5.35)	0.000
65–69	78	277	0.52 (0.40;0.63)	1.71 (1.51;1.91)	3.31 (2.57;4.25)	0.000
70–74	84	280	0.63 (0.49;0.76)	1.85 (1.63;2.07)	2.95 (2.31;3.77)	0.000
75–79	68	162	0.62 (0.48;0.77)	1.20 (1.02;1.39)	1.93 (1.45;2.56)	0.000
80–84	18	73	0.24 (0.13;0.34)	0.65 (0.50;0.80)	2.78 (1.66;4.65)	0.000
85–89	5	14	0.13 (0.02;0.24)	0.18 (0.09;0.27)	1.43 (0.52;3.98)	0.489
90 +	0	0	0.00 (0.00;0.00)	0.00 (0.00;0.00)	-	-
**Total**	**582**	**2,024**	**0.27 (0.25;0.29)**	**0.87 (0.83;0.91)**	**3.26 (2.97;3.58)**	**0.000**

## Discussion

PHPT is a prevalent endocrine disorder with a clinical presentation that has shifted significantly over the past three decades, largely due to greater access to routine biochemical screening. The proportion of asymptomatic patients has risen markedly, from 18% in the early 1960 s to over 80% today [9, 17, 18]. It is now well established that asymptomatic PHPT does not represent a homogeneous condition. According to the initial clinical presentation, patients can be classified into two categories, with possibly different natural histories and prognoses: those with evidence of target-organ involvement and those without [19]. Following the introduction of automated serum calcium measurement, multiple studies reported a rise in the incidence and prevalence of hypercalcaemia and hyperparathyroidism. Most researchers attributed this increase to improved case detection, which was consistent with the observation that the increased incidence was largely due to patients without overt complications of hyperparathyroidism and with only mildly elevated serum calcium levels [1, 6, 8, 9]. While the number of cases identified initially increased with the widespread adoption of automated calcium measurement, this was followed by a decline as the backlog was cleared—the so-called “catch-up” effect [6], observed largely in developed countries [8, 9].

In our region, the standardised incidence of PHPT has remained stable over the past decade.

A population-based study conducted in Rochester (Minnesota, USA), analysing PHPT incidence trends from 1965 to 2010, identified two peaks: the first coinciding with the introduction of automated calcium measurement panels in 1974, and the second occurring in the late 1990 s, following the publication of the first clinical practice guidelines on osteoporosis [8]. The latest catch-up phase has been attributed to increased awareness of bone fragility and osteoporosis, which led to a renewed rise in diagnostic evaluations of phosphate-calcium metabolism. After the catch-up phase, a long-term stabilization of incidence is expected, as reflected by the stable PHPT trend in our study.

However, it is not surprising that in our study the trend in males, although statistically stable, showed a tendency toward a decline, unlike in females. This may be explained by the continuous screening of female subjects for osteoporosis, including those who are far from menopause. In males, attention to the issue of osteoporosis is not as strongly felt.

Our study confirms that the peak incidence of PHPT in women corresponds to the postmenopausal period, a time when medical attention is frequently sought to monitor health status, leading inevitably to a peak in the diagnosis of PHPT, including mild forms. The post-menopausal PHPT incidence peak could also be related to pathophysiological reasons associated with the loss of the protective effect of oestrogen on bones [20]. The decline in oestrogen levels during menopause hastens bone loss, which can exacerbate or unmask primary hyperparathyroidism, intensifying the progression of bone disease. In men, the incidence peak occurs approximately 20 years later, probably when the individual comes to medical attention for other age-related health issues. Supporting the view that much of this post-menopausal peak in females is related to overdiagnosis is the analysis of incidence trends among only those patients who had undergone parathyroidectomy. According to the latest international as well as Italian guidelines on the management of primary hyperparathyroidism, although surgery is the definitive treatment in all cases, it is recommended primarily for patients presenting with signs of organ involvement associated with the disease, meaning the more severe cases, although the final decision should be assessed on a case-by-case basis, with consideration of patients’ preferences [21, 22]. The incidence curves by sex for operated patients appear to be much more parallel, and although the incidence remains higher in females at all ages, the peak is flattened. Furthermore, the median age at diagnosis for these patients is also comparable for the two sexes, unlike in the totality of cases, where it is significantly higher for males than females. This finding further supports the suggestion that a significant portion of the incidence in females is attributable to overdiagnosis of mild forms, and we cannot rule out that this may also explain the non-decreasing trend in females.

The earlier age peak in surgical cases compared with general cases is particularly interesting and the reason is not clear. The simplest explanation may be that asymptomatic cases require more time to come to the clinician's attention and thus to receive a diagnosis, in contrast to surgical cases, which generally present with more severe disease. Another explanation, although more speculative, may stem from the theory—although as yet unconfirmed—of two phenotypes of PHPT [1], which holds that patients with the type 1 phenotype would present with more severe disease, characterised by a shorter symptom duration, elevated calcium levels, advanced bone involvement, and larger tumours, while those with the type 2 phenotype would exhibit milder forms, with smaller tumours, lower calcium levels, and an asymptomatic course over a longer period. Emerging evidence indicates that a combination of factors—such as genetics and environmental influences—may underlie the diverse clinical presentations of PHPT [1]. In partial support of this hypothesis, it is worth noting that Saponaro et al., in a multicentric Italian study on the clinical presentation and management of PHPT, also found a lower age at diagnosis in cases cured with surgery compared with non-surgical cases. The former corresponded to the more severe cases in terms of albumin-adjusted serum calcium, PTH, urinary calcium excretion and 24-h urinary calcium levels [10].

Another noteworthy finding concerns the 25–29 age group, the only category in which males predominate (female-to-male ratio: 0.54). Although females slightly predominate in the preceding age group (18–24 years; female-to-male ratio: 1.07), the difference is not significant and as pronounced as in the older age groups, so we cannot exclude the possibility that we are looking at a diagnostic tail of paediatric PHPT cases. Such cases are often associated with a genetic background and with a female-to-male ratio that remains unclear—some studies have reported it skewed toward males [23, 24], others as balanced [25, 26]—but certainly lacking the clear female predominance observed at older ages.

Our study has both strengths and limitations. Among its strengths is the large, population-based dataset, which spans a lengthy observation period of over a decade. To the best of our knowledge, this is the only population-based study currently available in Italy on the incidence of PHPT. The main limitation is the lack of clinical and laboratory data, which precludes assessment of the severity of PHPT.

In conclusion, our study shows that over the past decade in our Region, there has been a general stability in the incidence of PHPT, although with a trend toward a decrease in males. A higher incidence was observed in postmenopausal females. When considering only surgical cases, which tend to be diagnosed at an earlier age than non-surgical cases, the incidence curves by sex confirm a female predominance, but the percentage distributions of incident cases by age show little difference between males and females.

## Supplementary Information

Below is the link to the electronic supplementary material.

Supplementary file1 (PNG 1002 KB)

## Data Availability

The data supporting the findings of this study are available on request from the corresponding author, Caterina Mian.
