# Supplementary figures and images for: Primary hyperparathyroidism incidence in northeastern Italy: A large population-based study
Source: J Endocrinol Invest. 2025 Oct 18;49(1):143–51. doi: 10.1007/s40618-025-02723-0 (PMC12847158; doi:10.1007/s40618-025-02723-0)

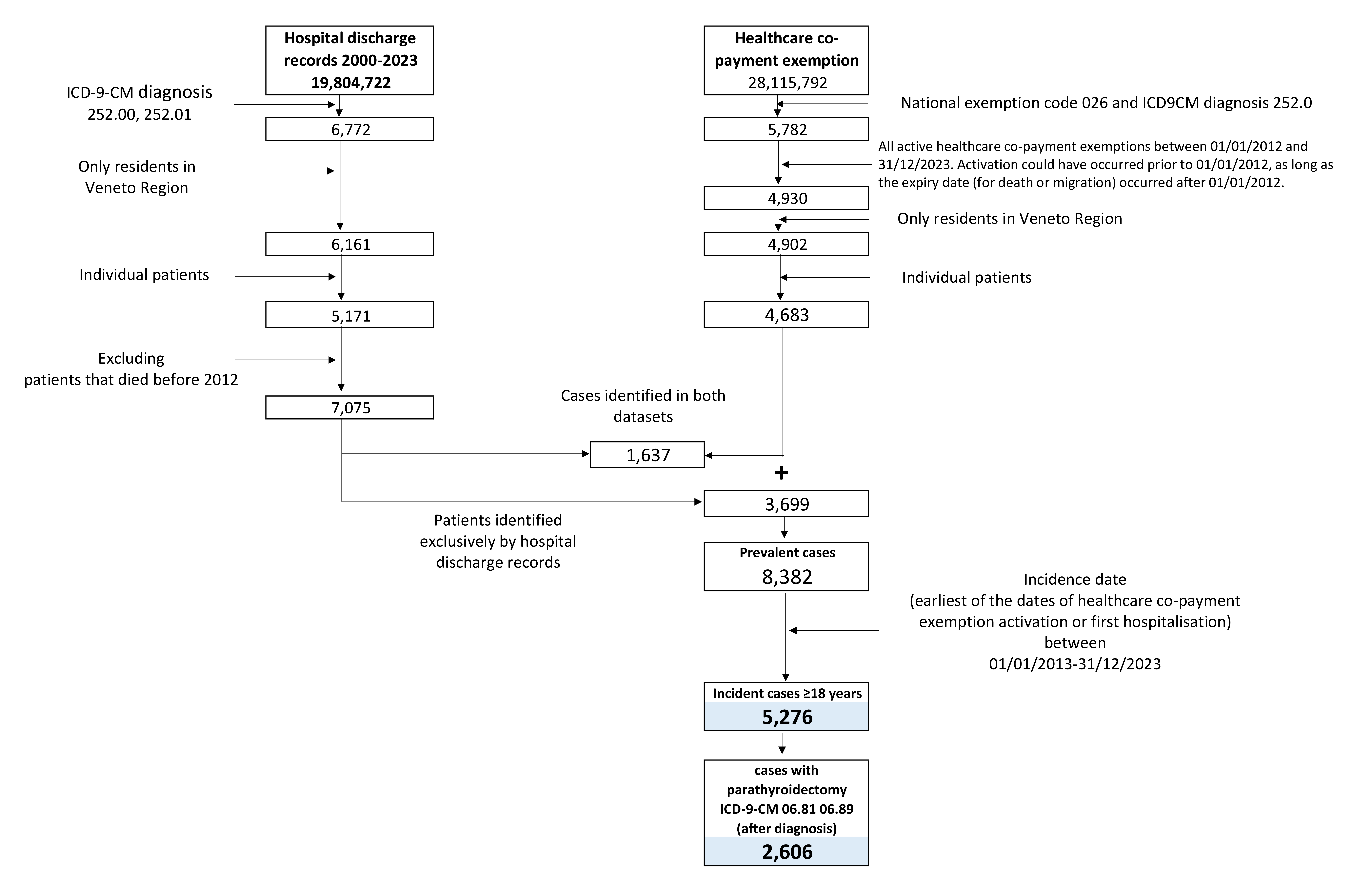

Supplement: Supplementary file 1 — Supplementary file1 (PNG 1002 KB) [file 40618_2025_2723_MOESM1_ESM.png]
